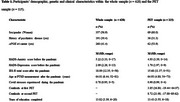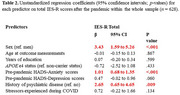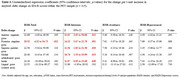# Amyloid accumulation within emotional regulation areas associated with PTSD symptomatology in cognitively unimpaired older adults 1,5 years after the Covid‐19 pandemic

**DOI:** 10.1002/alz70857_105045

**Published:** 2025-12-25

**Authors:** Eleni Palpatzis, Mahnaz Shekari, Muge Akinci, Juan Domingo Gispert, Karine Fauria, Anna Brugulat‐Serrat, Gonzalo Sánchez‐Benavides, Eider M Arenaza‐Urquijo

**Affiliations:** ^1^ Global Health Institute Barcelona (ISGlobal), Barcelona, Spain; ^2^ Barcelonaβeta Brain Research Center (BBRC), Pasqual Maragall Foundation, Barcelona, Spain; ^3^ Universitat Pompeu Fabra, Barcelona, Spain; ^4^ Barcelona Institute for Global Health (ISGlobal), Barcelona, Barcelona, Spain; ^5^ BarcelonaBeta Brain Research Center (BBRC), Barcelona, Spain; ^6^ Hospital del Mar Research Institute, Barcelona, Spain; ^7^ Centro de Investigación Biomédica en Red de Bioingeniería, Biomateriales y Nanomedicina (CIBER‐BBN), Madrid, Spain; ^8^ Barcelonaβeta Brain Research Center (BBRC), Barcelona, Spain; ^9^ Centro de Investigación Biomédica en Red de Fragilidad y Envejecimiento Saludable (CIBERFES), Madrid, Spain; ^10^ Centro de Investigación Biomédica en Red de Fragilidad y Envejecimiento Saludable (CIBERFES), 28089, Madrid, Spain; ^11^ ISGlobal ‐ Barcelona Institute for Global Health, Barcelona, Catalunya/Barcelona, Spain

## Abstract

**Background:**

Mental health symptoms are increasingly recognized as early manifestations of AD. However, the neurological basis for these associations is not well understood. We tested a regional vulnerability hypothesis by examining whether the rate of regional Aβ accumulation in areas affected early in AD and also involved in emotional processing was associated with post‐traumatic stress disorder (PTSD) symptomatology following the Covid‐19 pandemic.

**Method:**

We included 628 cognitively unimpaired individuals from the ALFA study with data available on post‐pandemic PTSD‐symptomatology (Impact of Events Scale Revised [IES‐R] Total, Intrusion, Avoidance and Hyperarousal scores) self‐reported 1.48 (SD=0.07) years after the Covid‐19 state of alarm ended in Spain. Additionally, 115 participants underwent two PET assessments ([^18^F] flutemetamol PET; mean time interval=3.36 years, SD=0.48). We calculated the Δ change in Aβ accumulation in pre‐defined ROIs (anterior cingulate gyrus, posterior cingulate gyrus, superior parietal gyrus, medial orbitofrontal gyrus, the insula and lingual gyrus [control area]). First, we performed linear regression models to examine whether sex/gender, education, *APOE* ε4‐status, pre‐pandemic anxiety and depression (Hospital Anxiety and Depression Scale; HADS), history of psychiatric disease and pandemic stressors were associated with total PTSD scores. Second, we performed linear regression models to examine the associations of Δ change in Aβ in ROIs with PTSD total and sub‐scores.

**Result:**

Participants’ age ranged between 52−81 and 57% were women (Table 1). Women, those with higher pre‐pandemic HADS‐anxiety scores and with a history of psychiatric disease reported higher total PTSD symptoms post‐pandemic (Table 2). Faster Aβ accumulation in the posterior cingulate gyrus, but no other ROIs, was associated with higher total PTSD symptoms post‐pandemic (Table 3). In terms of PTSD sub‐symptoms, a faster Aβ accumulation in all ROIs (except control area) was associated with intrusion symptoms, but not with avoidance or hyperarousal (Table 3).

**Conclusion:**

Our findings suggest that a faster rate of early Aβ accumulation in brain regions linked to emotional processing may heighten vulnerability to stress‐related symptoms. The association with intrusion symptoms across multiple regions underscores the role of dynamic changes in Aβ pathology in emotional regulation and rumination. Additionally, women and individuals with pre‐existing mental health symptoms appear more vulnerable.